# Aesthetic Rehabilitation of a Complicated Crown-Root Fracture of the Maxillary Incisor: Combination of Orthodontic and Implant Treatment

**DOI:** 10.1155/2014/925363

**Published:** 2014-04-29

**Authors:** Érica Dorigatti de Avila, Rafael Scaf de Molon, Mauricio de Almeida Cardoso, Leopoldino Capelozza Filho, Marilia Mattar de Amoêdo Campos Velo, Francisco de Assis Mollo, Luiz Antonio Borelli Barros

**Affiliations:** ^1^Department of Dental Materials and Prosthodontics, Araraquara School of Dentistry, São Paulo State University (UNESP), 1680 Humaita Street, 14801-903 Araraquara, SP, Brazil; ^2^Department of Diagnostic and Surgery, Araraquara School of Dentistry, São Paulo State University (UNESP), 1680 Humaita Street, 14801-903 Araraquara, SP, Brazil; ^3^Department of Orthodontics, University of Sagrado Coracao, 10-50 Irma Arminda Street, 17011-160 Bauru, SP, Brazil; ^4^Department of Physiological Sciences, Piracicaba Dental School, Campinas State University, 901 Limeira Avenue, 13414-903 Piracicaba, SP, Brazil; ^5^Department of Social Dentistry, Araraquara School of Dentistry, São Paulo State University (UNESP), 1680 Humaita Street, 14801-903 Araraquara, SP, Brazil

## Abstract

The aim of this paper is to present a complex rehabilitation, of fractured tooth, with implants in anterior region considering the orthodontics extrusion to clinical success. At 7 years old, the patient fractured the maxillary left central incisor and the dentist did a crown with the fragment. Twenty years later, the patient was referred to a dental clinic for orthodontic treatment, with the chief complaint related to an accentuated deep bite, and a professional started an orthodontic treatment. After sixteen months of orthodontic treatment, tooth 21 fractured. The treatment plan included an orthodontic extrusion of tooth 21 and implant placement. This case has been followed up and the clinical and radiographic examinations show excellence esthetic results and satisfaction of patient. The forced extrusion can be a viable treatment option in the management of crown root fracture of an anterior tooth to gain bone in a vertical direction. This case emphasizes that to achieve the esthetic result a multidisciplinary approach is necessary.

## 1. Introduction

Dental trauma occurs most commonly in the maxillary incisors, and most of the time, the dental crowns are damaged. In the presence of fractures, the treatment of teeth depends on the level of the fracture line, the type of occlusion [1, 2], and the prognosis [1]. Oblique fractures are more problematic to treat compared to horizontal fractures. At the same time, root fractures are also more likely to occur in fully erupted permanent teeth with closed apices, in which the totally formed root is firmly supported by bone and periodontal tissue [3]. When the fracture line is positioned below the alveolar bone crest, and if the apical root fragment is evaluated as being sufficient to support a coronal restoration, the coronal fragment can be removed and the root treated endodontically [4]. However, there are cases in which it is impossible to maintain the tooth, and the treatment requires a multidisciplinary approach to improve the functional and aesthetic outcomes [5]. The protocol indicated for these cases is extraction of the tooth, followed by implant rehabilitation. The possibility of rehabilitating the patient does not mean success of treatment.

To obtain the success of implant restoration in anterior aesthetic areas is necessary harmonious relationship between the implant and peri-implant tissue and the remaining natural teeth [5]. Although the techniques and materials that have been developed are capable of high success rates to achieve excellent aesthetic results, it is important that the bone and gingival level be in accordance with the adjacent bone [6]. Hence, bone grafting could be a prerequisite for successful implant dentistry in select cases. Another important alternative to bone grafting is orthodontic extrusion [7, 8]. Several authors have indicated orthodontic extrusion for vertical bone gain [7–11]. The main objective of this procedure is both to provide a tissue margin for the final restoration and to create a periodontal environment (biologic width) that is easy to maintain [7, 9, 11]. According to H. Salama and M. Salama [10], orthodontic eruption should be used to create at least 2 mm of additional vertical gingival tissue relative to adjacent teeth, to produce a harmonious gingival arrangement. Once peri-implant harmony is restored, the results with an aesthetic and functional prosthesis will have greater predictability. This paper presents a complex case of rehabilitation, where it was a considerate orthodontics extrusion and a combined surgical technique to obtain the clinical success.

## 2. Case Report 

In 1990, a 7-year-old white female suffered a trauma in a swimming pool and fractured the crown of her maxillary left central incisor. Her parents found the fragment, and a pediatric dentist placed a crown with the autogenous fragment. The fractured line of the left maxillary central incisor was below the buccal gingival margins, restricted to the crown. Radiographic examinations showed no abnormality; the tooth was followed up for a few months (clinical and radiographic controls) and showed no signs or symptoms that could lead to suspicion of pulp necrosis.

**Figure 1:**
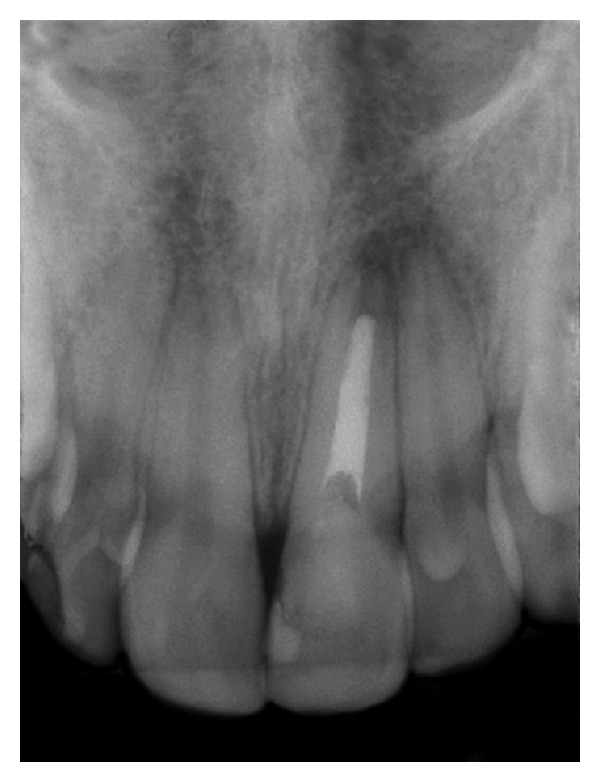
Radiographic view of the fractured tooth.

**Figure 2:**
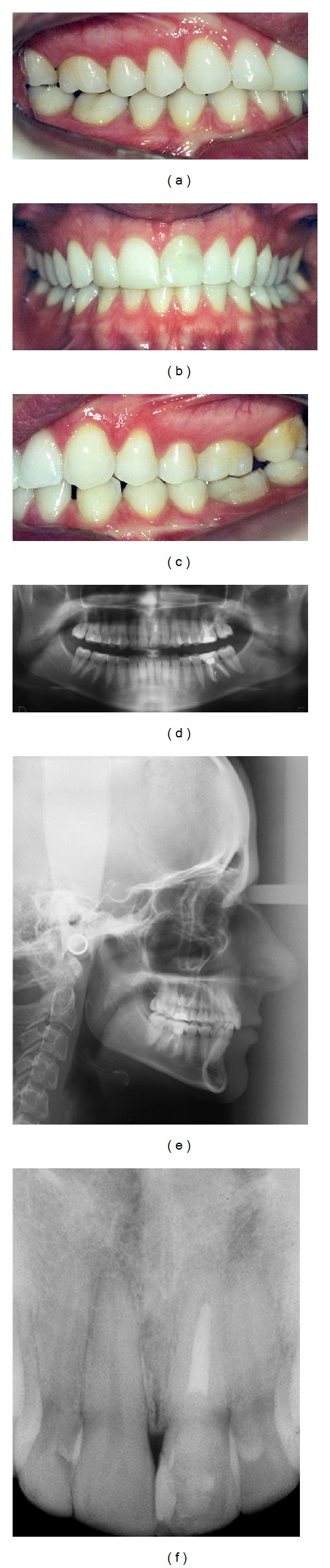
(a, b, c) Preoperative views; (d) panoramic radiography; (e) cephalometric radiography; (f) periapical radiography showing no significant changes to the maxillary left central incisor.

At eleven years old, while on a school trip, the patient developed swelling and drainage in her anterior maxilla, and she was immediately referred to an endodontist for root canal treatment of the maxillary left central incisor. The treatment was limited regarding pulp calcification; the obturator material did not fill in to complete the canal in the apical region (Figure 1). At sixteen years old, the maxillary left central incisor darkened, and the patient underwent intracanal whitening. Eleven years later, at 27 years old, the patient was referred to a dental clinic for orthodontic treatment, with the chief complaint related to an accentuated deep bite, and a professional started orthodontic treatment. At this time, the patient presented a Class I occlusal relationship in the right side, a Class II full occlusion on the left side (Figures 2(a) to 2(c)), an overbite and opened interincisor angle, a history of clenching, and TMD signs (click at both ATMs). At this stage, a periapical radiograph showed no significant changes in comparison to X-rays taken immediately after the root canal of the maxillary left central incisor (Figures 2(d) to 2(f)).

**Figure 3:**
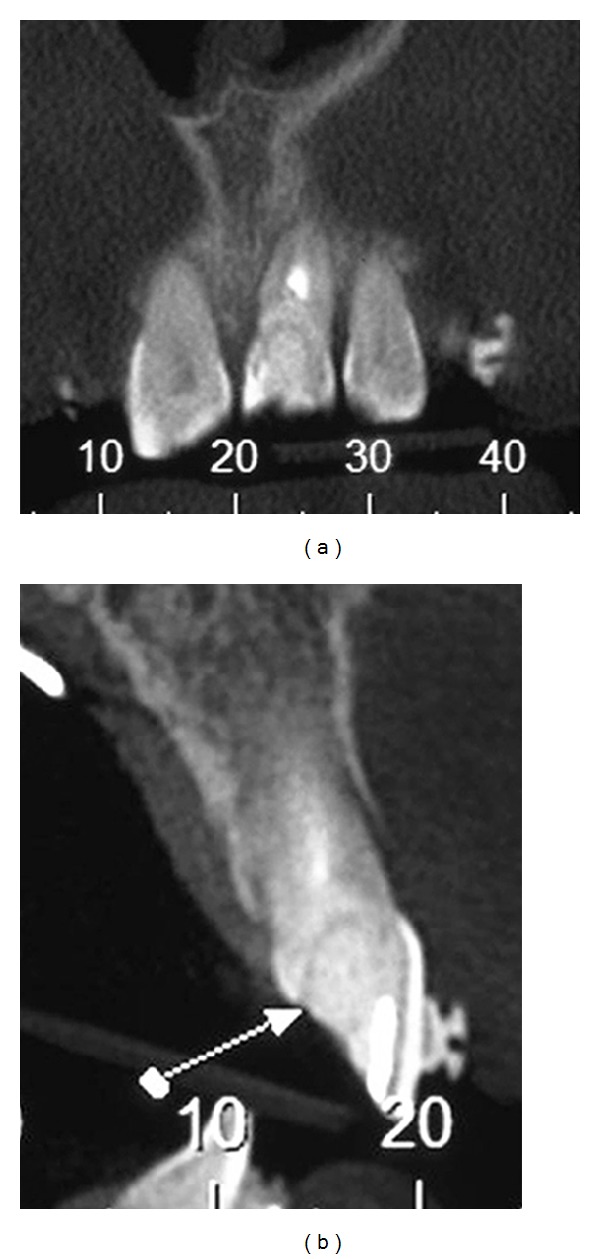
Computerized tomography showing the fracture line.

After sixteen months of orthodontic treatment, the maxillary left central incisor fractured. The fracture was confirmed by means of Cone Beam Computed Tomography (Figures 3(a) and 3(b)). The patient did not agree with the treatment plan of this professional, which involved immediate extraction of maxillary left central incisor with implant placement, and she sought another professional opinion. At this time, our team evaluated the patient, and the prognosis at this stage was good for compensatory treatment (interincisor angle closure) and questionable for the maxillary left central incisor, requiring mechanical extrusion to expose the fracture line and reassessment to maintain the root or removal for implant placement. It is important to emphasize that the patient is a dentist, and she wanted to participate in the elaboration of treatment plan. She did not agree to have the tooth extracted—despite of the tooth presenting periapical lesions—without an attempt to keep the tooth and to undergo endodontic surgery at a later time for lesion removal. Thus, the conservative treatment plan included orthodontic treatment, including forced orthodontic extrusion of the maxillary left central incisor until exposure of the fracture line to allow for the fabrication of a crown.

**Figure 4:**
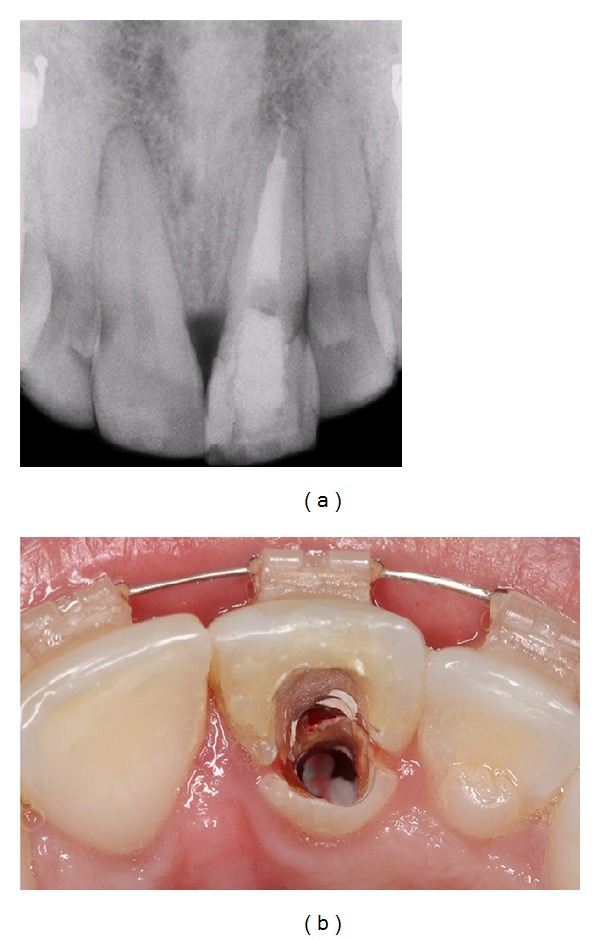
Radiographic and clinical images showing the endodontic retreatment, with the installation of an intracanal pin and temporary crown placement.

**Figure 5:**
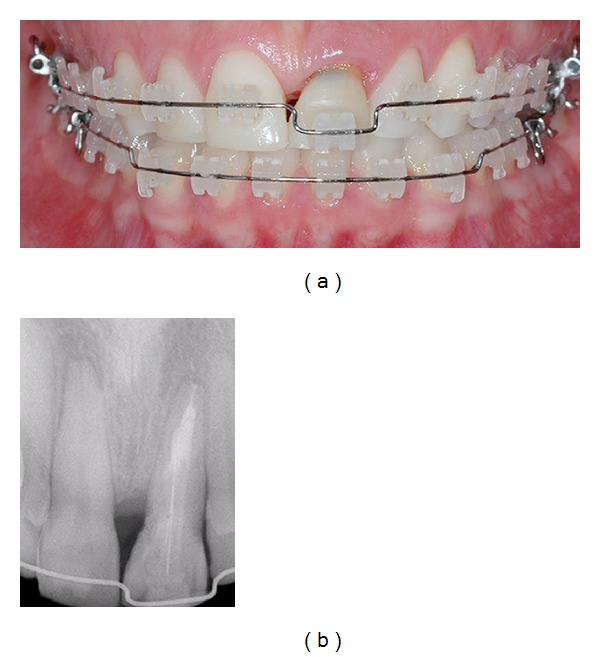
(a, b) Radiographic and clinical images illustrating orthodontic extrusion.

At this time, endodontic retreatment was performed, with installation of an intracanal pin (Figures 4(a) and 4(b)). The purpose of the pin was only to strengthen the tooth to allow for orthodontic extrusion and consequently to gain bone. Subsequently, forced orthodontic extrusion of the central incisor was undertaken (Figures 5(a) and 5(b)) to restore the physiological periodontal attachment, according to de H. Salama and M. Salama [10]. The maxillary and mandibular arch were leveled using a stainless steel .018” arch wire, combined with bending for the extrusion of tooth 21, and each return was pronounced to achieve tooth extrusion.

**Figure 6:**
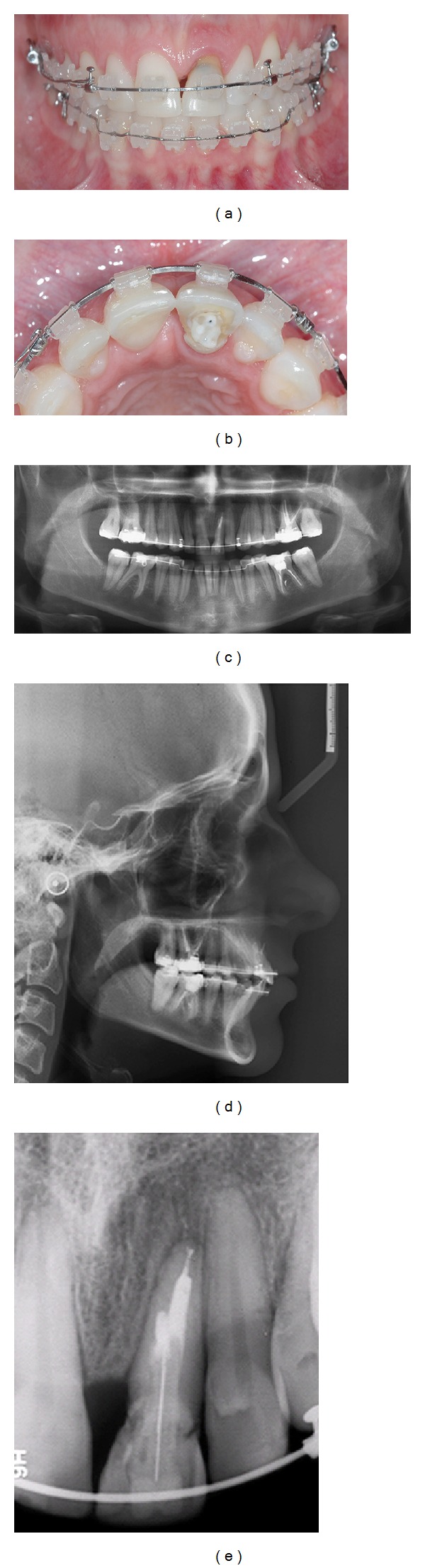
(a, b) Clinical images illustrating orthodontic extrusion after eight months; (c) panoramic radiography; (d) cephalometric radiography; (e) periapical radiography.

Concomitant to the tooth traction, Class II asymmetric mechanics were performed with elastics for the correction of the sagittal relationship, corroborating interincisor angle closure and lower midline and overbite correction. The orthodontic extrusion was completed after 8 months, and the maxillary left central incisor was repositioned and fixed with an orthodontic appliance in the cervix, with the intention of stabilizing the carried motion. At this stage, with the goal of orthodontic treatment achieving an end, orthodontic documentation was requested, and the lower braces were removed (Figures 6(a) to 6(e)).

**Figure 7:**
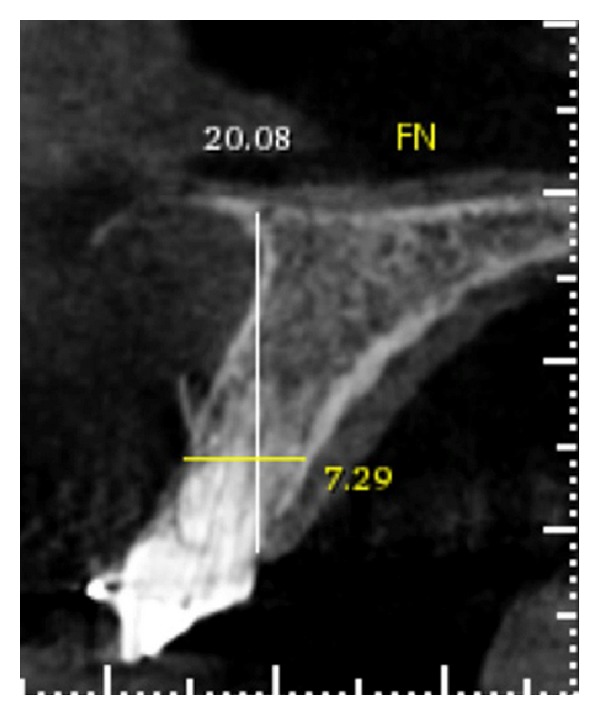
Computed tomography after orthodontic extrusion. Perforation was observed in the root.

Computed tomography (CT) was obtained to reevaluate the response of tooth 21 after orthodontic extrusion, and a perforation in the root was observed, likely a consequence of successive attempts at endodontic retreatment (Figure 7). At this time, the team decided on implant placement with bone grafting, connective tissue grafting, and prosthesis.

**Figure 8:**
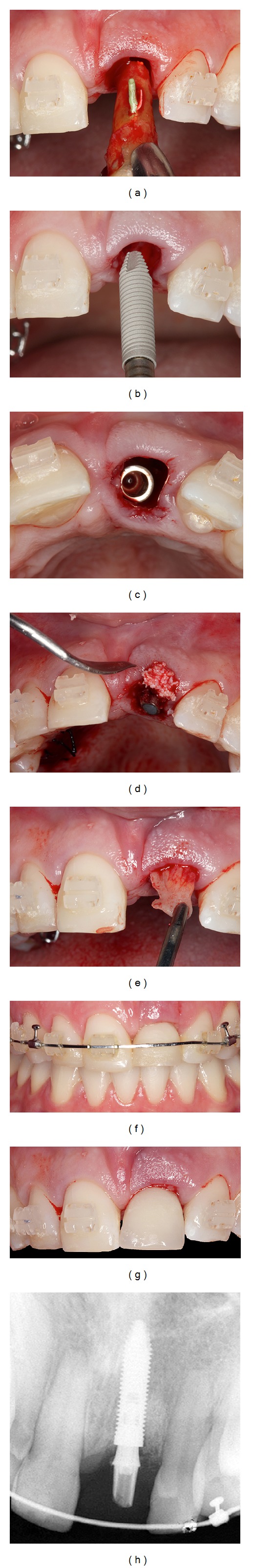
(a) Extrusion of the tooth; (b) implant placement; (c) position of the implant. (d) Bone graft with Bio-Oss; (e) subepithelial connective tissue graft inserted to improve the periodontal tissue biotype. (f) Radiographic examination showing the implant; (g), (h) provisional crown performed in infraocclusion.

The implant procedure was planned considering the bone gain obtained with mechanical extrusion and the level of exposure during smiling. The surgical procedure was initiated by incision of the buccal and lingual soft tissues around the tooth to be extracted, as well as on the contralateral aspect of each adjacent tooth (Figure 8(a)). Full-thickness flaps were elevated, and a minimally invasive surgery was performed to preserve the alveolar bone, using a periotome (Hu-Friedy, Chicago, IL, USA), associated with curettage of the periapical lesion. The treatment succeeded with implant placement (Figure 8(b)).  Figure 8(c) shows the implant position (Figure 8(c)). After implant placement, an exact universal abutment (Morse Taper) was installed (Neodent, Curitiba, PR, Brazil). The provisional abutment was realigned and was adapted on the sleeve prior to cementing, and the gap between the bone and the implant labial plate was filled with Bio-Oss (Geistlich, Wolhusen, Switzerland) (Figure 8(d)). A subepithelial connective tissue graft (Figure 8(e)) was inserted to improve the periodontal tissue biotype [12], thus increasing the range of keratinized gingiva, favoring the red aesthetics, and avoiding recession. After implant installation, a provisional crown was performed in infraocclusion (Figures 8(f) to 8(h)).

**Figure 9:**
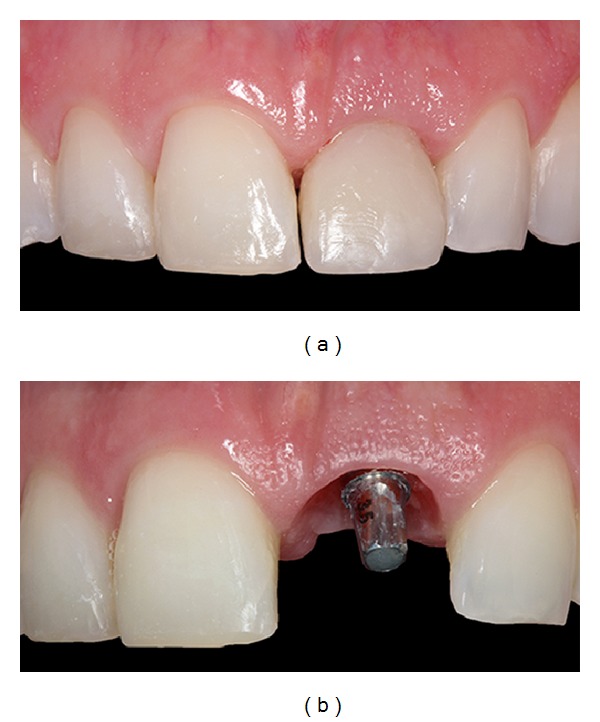
Images showing the provisional crown, restoring the maxillary right central incisor and gingival tissue after four months.

**Figure 10:**
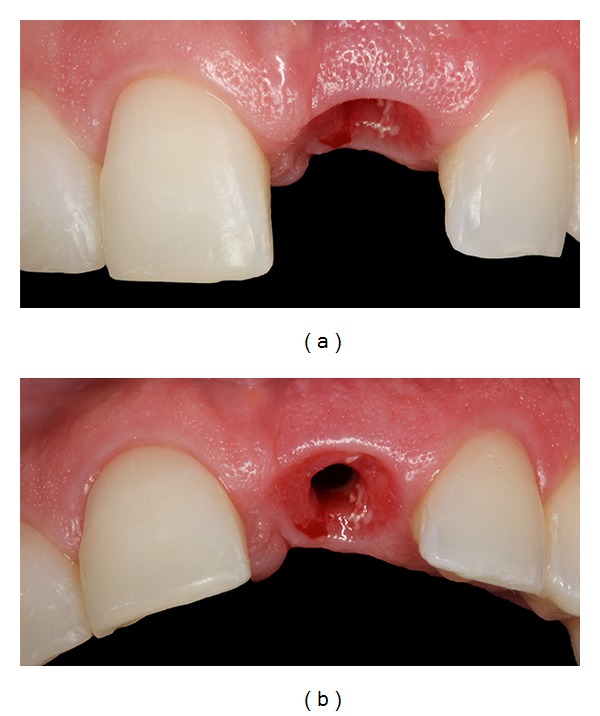
The gingival color, texture, and contour appeared similar to the adjacent soft tissues of the teeth.

**Figure 11:**
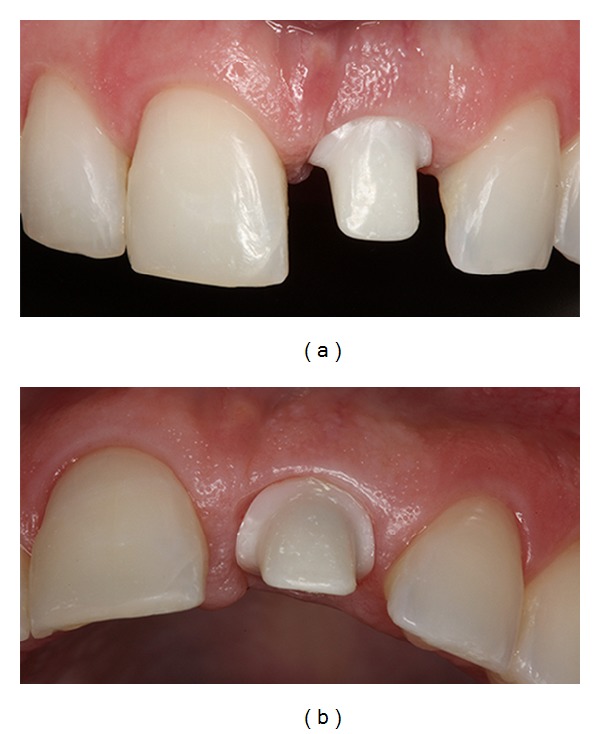
Buccal and occlusal views, showing the custom abutment in zirconia with a CAD/CAM system.

**Figure 12:**
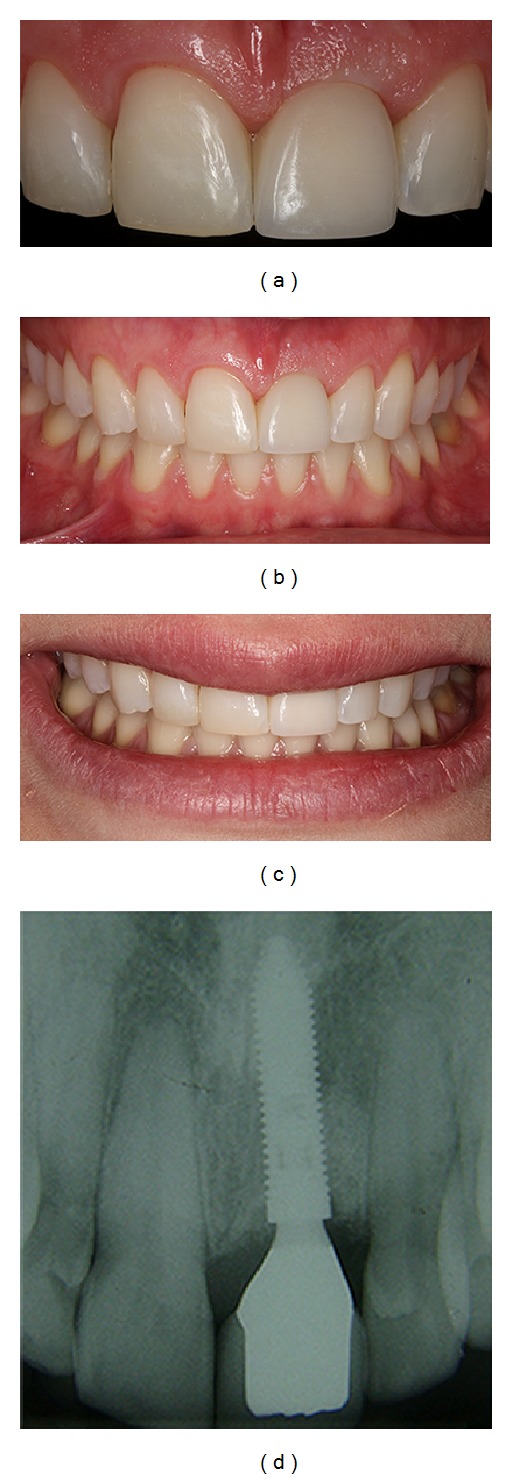
(a, b, c, d) Postoperative clinical and radiographic views, immediately after the conclusion of the prosthetically restored maxillary right central incisor.

**Figure 13:**
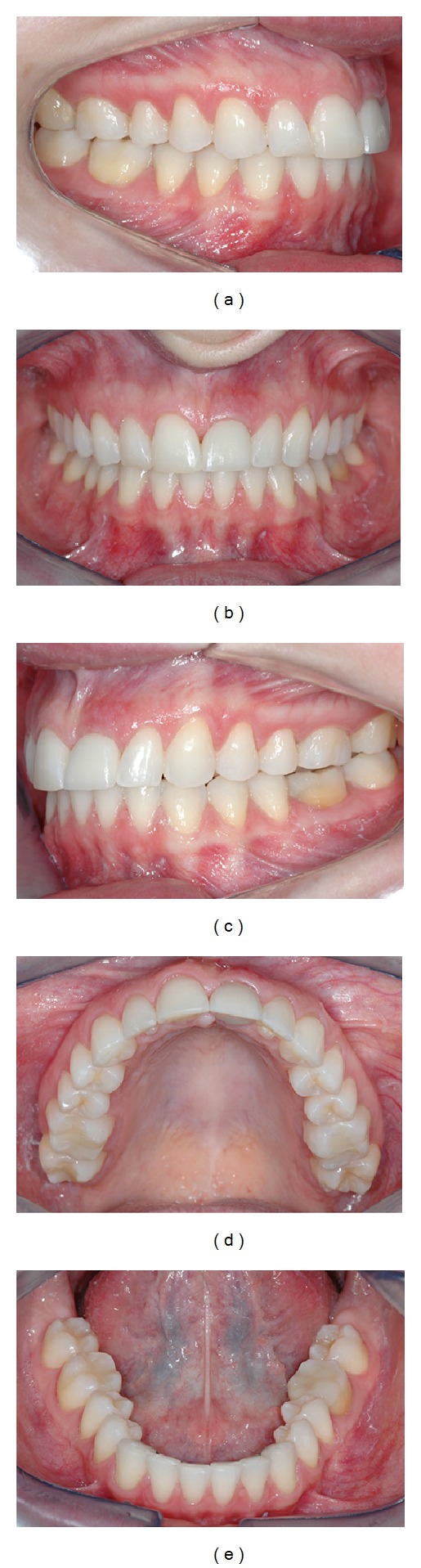
Follow-up clinical views (lateral, central, and occlusal) after 1 year.

Four months later, the provisional crown was removed to evaluate the gingival tissue (Figures 9(a) and 9(b)). The gingival color, texture, and contour appeared similar to the adjacent soft tissues of the teeth (Figures 10(a) and 10(b)). The definitive prosthesis was created eight months after the implant surgery, with implant and peri-implant tissue impressions obtained to create a custom abutment in zirconia with a CAD/CAM system (Figures 11(a) and 11(b)). This case has been followed up, and the clinical and radiographic examinations have shown excellent aesthetic results (Figures 12(a) to 12(d)) and satisfaction of the patient. Clinical success was achieved in this case with multidisciplinary treatment after 1-year follow-up (Figures 13(a) to 13(e)).

## 3. Discussion

Dental trauma in young patients, mainly to the permanent central incisor, can cause aesthetic and functional problems, which in turn can result in severe emotional problems [13]. In this paper, we reported a rehabilitation of a complex case of fractured maxillary central incisor tooth in which, among the options treatment, we opted for orthodontic extrusion to bone formation and implant rehabilitation.

Despite possible contamination by saliva via the fracture line into the pulp, teeth normally remain free of symptoms, as in the case in question. However, definitive treatment should begin within a few days after the trauma, contrary to what occurred with our patient. In this case, the first professional did not treat the tooth canal, and the patient was only examined by a new dentist some years later when she felt pain, and edema appeared. The following situations must be considered before choosing the treatment plan: the localization and degree to which biologic width has been invaded; the presence or absence of pulpal involvement; the root development stage; the tooth eruption stage; and the degree of adaptation of the fragment to the tooth remnant.

Extraction must not be the first treatment choice for fractured teeth in cases of young patients with permanent teeth in the anterior region for several reasons [4]. Depending on the fracture site, some clinical procedures might be performed to save the tooth; if it is not possible to keep the tooth in the arcade, the tooth can be used during extrusion to stimulate bone formation in the vertical direction and to facilitate the implant installation. Multidisciplinary treatment allows patients with traumatized anterior teeth to be rehabilitated with success. In the case in question, success was obtained with a combined approach involving immediate flapless implant placement, connective tissue, and bone graft followed by immediate provisionalization of the crown as described previously by de Molon et al. [14].

In the case of aesthetic areas, the challenge of rehabilitation is even greater. Dental implants have significantly favored clinical results when the patient has lost a tooth [15, 16]. However, to obtain aesthetic results, the presence of bone in both directions is necessary. New bone formation in defects, caused by periodontal disease, trauma, or alveolus postextraction, requires three fundamental factors: the presence of a blood clot, preserved osteoblasts, and contact with living tissue [17]. New bone formation can be obtained through bone grafting or, in some cases, orthodontic extrusion. In the case in question, we opted for orthodontic extrusion. After the decision was made to extract the tooth, all the procedures were performed in an attempt to maintain the tooth responsible for eruption.

Forced orthodontic extrusion could be an appropriate adjunct for aesthetic and functional restorations because the attachment apparatus of natural teeth maintains and augments the shape of the alveolar bone, as well as the gingival and alveolar mucosa [18, 19]. H. Salama and M. Salama [10] showed that orthodontic extrusion of teeth with advanced periodontal disease could help to eliminate deep infrabone defects and might allow for more optimal implant placement, with a higher level of aesthetic predictability. Some years later, these authors suggested that improvements in the height and position of the interproximal bone worked effectively to achieve normal interdental papillary heights [6].

The clinical and radiographic examinations of our patient showed bone gain in the vertical direction, allowing for implant placement in an ideal 3-dimensional position. The particulate bone graft was inserted in the buccal side due to bone dehiscence, as well as a connective tissue graft to improve the red aesthetics. This case shows that clinicians should therefore always consider the appropriate timing of procedures to prevent added clinical difficulty.

In conclusion, this case report shows that extrusion can be a viable treatment option in the management of crown-root fracture of permanent anterior teeth, to gain bone in the vertical direction before tooth extraction and implant placement. At the same time, this case emphasizes that, to achieve aesthetic results, a multidisciplinary approach is necessary.
